# DNAzyme-mediated recovery of small recombinant RNAs from a 5S rRNA-derived chimera expressed in *Escherichia coli*

**DOI:** 10.1186/1472-6750-10-85

**Published:** 2010-12-06

**Authors:** Yamei Liu, Victor G Stepanov, Ulrich Strych, Richard C Willson, George W Jackson, George E Fox

**Affiliations:** 1 Department of Biology and Biochemistry, University of Houston, Houston, TX, USA; 2 Department of Chemical and Biomolecular Engineering, University of Houston, Houston, TX, USA; 3 BioTex, Inc, Houston, TX, USA

## Abstract

**Background:**

Manufacturing large quantities of recombinant RNAs by overexpression in a bacterial host is hampered by their instability in intracellular environment. To overcome this problem, an RNA of interest can be fused into a stable bacterial RNA for the resulting chimeric construct to accumulate in the cytoplasm to a sufficiently high level. Being supplemented with cost-effective procedures for isolation of the chimera from cells and recovery of the recombinant RNA from stabilizing scaffold, this strategy might become a viable alternative to the existing methods of chemical or enzymatic RNA synthesis.

**Results:**

Sequence encoding a 71-nucleotide recombinant RNA was inserted into a plasmid-borne deletion mutant of the *Vibrio proteolyticus *5S rRNA gene in place of helix III - loop C segment of the original 5S rRNA. After transformation into *Escherichia coli*, the chimeric RNA (3×*pen *aRNA) was expressed constitutively from *E. coli rrnB *P1 and P2 promoters. The RNA chimera accumulated to levels that exceeded those of the host's 5S rRNA. A novel method relying on liquid-solid partitioning of cellular constituents was developed for isolation of total RNA from bacterial cells. This protocol avoids toxic chemicals, and is therefore more suitable for large scale RNA purification than traditional methods. A pair of biotinylated 8-17 DNAzymes was used to bring about the quantitative excision of the 71-nt recombinant RNA from the chimera. The recombinant RNA was isolated by sequence-specific capture on beads with immobilized complementary deoxyoligonucleotide, while DNAzymes were recovered by biotin affinity chromatography for reuse.

**Conclusions:**

The feasibility of a fermentation-based approach for manufacturing large quantities of small RNAs *in vivo *using a "5S rRNA scaffold" strategy is demonstrated. The approach provides a route towards an economical method for the large-scale production of small RNAs including shRNAs, siRNAs and aptamers for use in clinical and biomedical research.

## Background

In recent years, small RNAs, including siRNAs (small interfering RNAs), shRNAs (small hairpin RNAs), aptamers, and ribozymes [1-9] have attracted increasing interest for their fundamental role in gene regulation, as well as for the potential of their use as novel diagnostic and therapeutic agents [10-16]. Interfering RNAs have generated particular interest due to its ability to effectively silence genes. For example, large-scale RNAi screens have been conducted to identify important genes in various biological pathways [17]. Multiple siRNA-based therapies are currently under development and soon may be used in the treatment of diseases such as hepatitis virus infection, macular degeneration, leukemia, and acquired immune deficiency syndrome [18-21]. Likewise, due to their high affinity and relatively low cost, aptamers have been used in numerous investigations seeking novel diagnostic tools or new drugs [7,22-25]. For example, an anticoagulant RNA aptamer that specifically binds and inactivates Factor IXa, is currently in Phase II clinical trials [23,26]. With the expanding use of small RNAs in basic and applied biological research, the demand for large quantities of synthetic RNAs of high quality has dramatically increased. Traditional methods of chemical and enzymatic synthesis are effective but are currently very expensive when large amounts are needed. Thus, a cost-effective method to produce defined small RNAs in large quantities is urgently needed.

The *in vivo *expression of recombinant RNAs has been described for some time [27-29]. However, the heterogeneity of the RNA products and their instability in the cytoplasm due to cleavage by cellular ribonucleases (RNases) have made the process inefficient. For example, mRNAs expressed in *E. coli *from T7 promoter exhibit a substantial heterogeneity due to ambiguous termination of the RNA transcripts [28,29]. Attempts to obtain *in vivo *expression of aptamers also were not successful, apparently because the resulting transcripts were again heterogeneous and did not accumulate to any substantial level [30]. One way to overcome these limitations is by expressing RNA molecules of interest in a tRNA scaffold [30,31]. In one such study, the epsilon sequence of human hepatitis B virus (HBV) was inserted into cloning sites surrounded by a tRNA^Lys ^or tRNA^Met ^scaffold under the control of *E. coli lpp *promoter. The tRNA scaffold formed a structure that protected the RNA insert from nucleolytic digestion. The tRNA-HBV chimera was shown to be successfully expressed in *E. coli *and could be extracted by affinity capture when the chimeric RNA contained in addition a sephadex or streptavidin aptamer module. The RNA insert was released from the tRNA scaffold using the enzyme RNase H and two unmodified guide deoxyoligonucleotides. However, the reported absolute positional specificity of RNA cleavage by RNase H is uncommon under the described conditions [32-34], and cannot be regarded as a general case. In addition, it is not yet known if a large variety of RNAs can be successfully prepared with this system or how the presence of the construct may affect the host cell.

**Figure 1:**
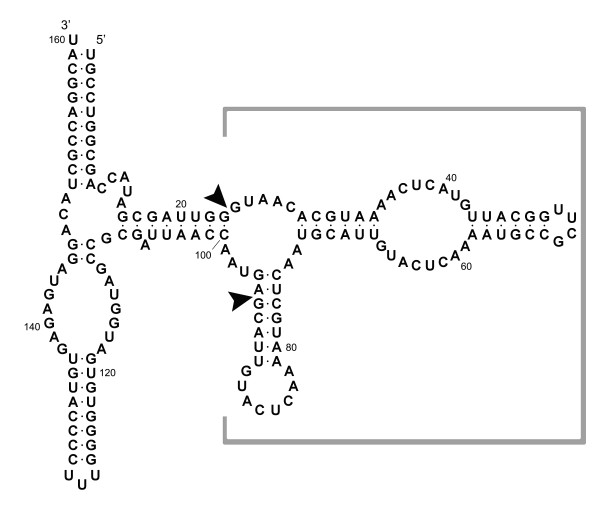
**Sequence and predicted secondary structure of 3×*pen *aRNA (160 nt)**. Sequence outside the grey box is the 5S rRNA scaffold, *Bst *aRNA [35], and sequence inside the grey box is RNA insert (figure modified from [37]). DNAzymes Pen17zyme1, -1B, and -1C are expected to cut 3×*pen *aRNA after nucleotide 94, and Pen17zyme2 is expected to cut after nucleotide 23. The excision sites on 3×*pen *aRNA are marked by arrows, and the expected excision product is 71 nucleotides long.

Herein, a fermentation-based system for expressing and purifying functional RNAs, especially RNAs of less than 100 nucleotides, is described. In this system, RNA sequences of interest are expressed under the strong P1 and P2 ribosomal promoters from the *E. coli rrn*B operon in the context of a carrier derived from 5S rRNA [35]. The resulting RNA product does not enter the ribosome but nevertheless accumulates to levels comparable to those of wild type 5S rRNA. In previous work with this system, various RNA insert sequences were incorporated into the plasmid-encoded 5S rRNA scaffold [36,37]. In each case, the expressed RNA/insert chimeras accumulated to high levels in the cell. An examination of the transcriptome revealed that the presence of the insert had essentially no effect on gene expression in the host cell [38]. In order for this approach to be broadly applicable, however, it will be necessary to extract the RNA of interest from the 5S rRNA carrier. We demonstrate herein that this can be effectively accomplished using sequence-specific DNAzymes [39-43]. An RNA substrate known as the 3×*pen *aRNA [37] (Figure 1) is used as a model system. The insert in this case is highly structured, as are many of the RNAs of interest and, at 71 nucleotides, illustrates that relatively large RNAs can be expressed in the 5S rRNA carrier system with good success.

## Methods

### Chemicals

Enzymes were purchased from Promega (Madison, WI) or New England Biolabs (Ipswich, MA). Synthetic deoxyoligonucleotides (Table 1) were obtained from Eurofins MWG (Huntsville, AL) and IDT (Coralville, IA). DNA markers were purchased from NEB and Bionexus (Oakland, CA). Chemicals were obtained from Sigma (St. Louis, MO) and EMD Chemicals (Gibbstown, NJ).

**Table 1:** Sequences and modifications of deoxyoligonucleotides used in this study

Name of oligos	Sequence (from 5' to 3')	Length (nt)
Pen17zyme1	CGCTAATTGGTTA**TCCGAGCCGGTCGAA**CGTAACATGAGTT	41

Pen17zyme1B	GCTAATTGGTTAC**TGTCAGCGACACGAA**GTAACATGAGTTTTAC	44

Pen17zyme1C	CGCTAATTGGTTAGT**CAGCTGACTCGAAC**GTAACATGAGTTTTAC	45

Pen17zyme2	GAGTTTTACGTGTTA**TGTCAGCGACACGAA**CAATCGCTATGGTC	44

bioPEN17zyme1B	Biotin-GCTAATTGGTTAC**TGTCAGCGACACGAA**GTAACATGAGTTTTAC	44

bioPEN17zyme2	Biotin-GAGTTTTACGTGTTA**TGTCAGCGACACGAA**CAATCGCTATGGTC	44

bioantiPEN	Biotin-GAGTTTTACGAGTTACGTAACATGAGTTTTAC	32

Underlined bold letters indicate residues that comprise the catalytic core of each DNAzyme

### RNA expression scaffold

Plasmid pCP3×3 [37] was electroporated into *E. coli *JM109 (DE3) (Promega) and used as the RNA expression scaffold in this work. The core element of the plasmid is the truncated 5S rRNA gene from *Vibrio proteolyticus *with a 71-nt insertion sequence. Gene expression is controlled by the *E. coli rrnB *P1 and P2 promoters. 3×*pen *aRNA coding sequence is followed by the *E. coli rrnB *T1 and T2 transcription terminators.

### Isolation of small RNAs from *E. coli*

Cells were grown at 37°C in LB medium with constant shaking (250 rpm). 100 μg/ml ampicillin was added to plasmid-carrying cells. Cell growth (OD_600_) was monitored using a Labomed Spectro SC spectrophometer. After incubation, cells were collected by centrifugation (30 min, 5000 × g, 4°C). The resulting (typically 200 mg) pellet was resuspended in 960 μl of 10 mM Tris-HCl (pH 7.5). Cells were lysed by adding 160 μl of 1 M Tris-Acetate with 0.1 M EDTA (pH 7.5), 160 μl of 10% SDS and 320 μl of pure formamide. After 20 min shaking at 37°C, 1.6 ml of 3 M potassium acetate (pH 4.8) was added, and the mixture was shaken gently for another 10 min. Cell debris and precipitated material were removed by centrifugation (20 min, 10000 × g, 4°C). The cleared supernatant was recovered and mixed with 8 ml of ethanol. After 1 h incubation at -80°C, the precipitated nucleic acids were collected by centrifugation (20 min, 10000 × g, 4°C). The pellet was then resuspended in 300 μl of 3 M sodium acetate (pH 5.0), and was shaken at 37°C for 10 min to selectively solubilize the small RNAs. Insoluble material was removed by centrifugation (20 min, 10000 × g, 4°C), and the supernatant was mixed with 750 μl of ethanol. Finally, the precipitate containing mostly small RNAs was collected by centrifugation (20 min, 10000 × g, 4°C), washed twice with 70% ethanol, and air dried for 15 min.

RNA samples were analyzed by gel electrophoresis and staining as previously described [44,45]. Low Molecular Weight DNA Ladder (New England Biolabs, Ipswich, MA) was used as molecular weight standards. The ratio of 3×*pen *aRNA accumulation level to that of 5S rRNA was determined using the freely available software package, ImageJ [46].

### Preparative PAGE

The 3×*pen *aRNA was purified to homogeneity by electrophoresis on an 8% preparative denaturing polyacrylamide gel. After separation, RNA bands were visualized by UV shadowing over Silica F254 TLC plates (Whatman), and excised using a sterile blade. RNA was eluted from gel slices by triple extraction with equal volumes of 50 mM HEPES-NaOH (pH 7.5), 1 mM EDTA, 150 mM NaCl, 19.2 M formamide. RNA was precipitated from the collected extracts by mixing with 0.1 vol. 3 M sodium acetate (pH 5.0) and 2.5 vol. ethanol. The sample was centrifuged (20 min, 10000 × g, 4°C), the collected precipitate was washed twice with 70% ethanol, and air dried for 15 min. Dry RNA pellets were dissolved in water, and their concentration was determined spectrophotometrically.

### Cleavage with DNAzymes

The DNAzymes used for the excision of RNA fragments from the 5S rRNA scaffold are listed in Table 1. The reaction conditions screened during optimization of 3×*pen *aRNA cleavage are summarized in Table 2. DNAzymes were annealed to the 3×*pen *aRNA substrate in 50 mM MOPS-NaOH (pH 7.2) containing additional components as specified in Table 2, at 90°C for 2 min, and then cooled to 23°C over 10 min in a thermal cycler (Eurofins-MWG). After annealing, the mixture of DNAzymes and aRNA was adjusted to contain 125 mM KCl, 500 mM NaCl, 7.5 mM MgCl_2_, MnCl_2 _(up to 45 mM), and 50 mM MOPS (pH 7.2) besides the indicated additional components, and incubated at 23°C or 40°C for up to 72 hours. In reaction 16 and 23, 50 and 6 additional cycles were introduced, respectively. After the incubation, the reaction was terminated by adding 0.1 vol. 3 M sodium acetate (pH 5.0). The reaction products were recovered by ethanol precipitation and analyzed by denaturing PAGE. The intensity of bands in these reactions was plotted using the 'profile' function of ImageJ [46], and the total cutting percentage was quantified as P = 1 - [intact 3×*pen *aRNA]/[total 3×*pen *aRNA]. Similarly, ImageJ was used to determine the yield of final product relative to the starting amount of chimeric aRNA per wet cell paste.

**Table 2:** Conditions of 3×pen aRNA cleavage evaluated during optimization of the reaction yield.

**Reaction No**.	[3×*pen *aRNA] (μM)*	Molar ratio 3×*pen *aRNA: Pen17zyme1/1B/1C: Pen17zyme2	Additional components in the annealing buffer	[Mn^2+^] (mM)*	Reaction T (°C)	Time	Additional Cycles
1	0.25	1:0:1	N/A	0	23	10 min	0

2	0.25	1:0:1	N/A	7.5	23	10 min	0

3	0.25	1:0:1	N/A	15	23	10 min	0

4	0.25	1:0:1	N/A	45	23	10 min	0

5	0.25	1:0:1	N/A	15	23	100 min	0

6	0.25	1:1(Pen17zyme1):0	N/A	15	23	100 min	0

7	0.25	1:1(Pen17zyme1B):0	N/A	15	23	100 min	0

8	0.25	1:1(Pen17zyme1C):0	N/A	15	23	100 min	0

9	0.25	1:5(Pen17zyme1B):0	N/A	15	23	100 min	0

10	0.25	1:1(Pen17zyme1B):0	N/A	15	23	17 h	0

11	0.25	1:1(Pen17zyme1B):0	N/A	15	40	100 min	0

12	0.25	1:1(Pen17zyme1B):0	10%EtOH	15	23	100 min	0

13	0.25	1:5(Pen17zyme1B):0	0.3 M KCl, 1.2 M NaCl	15	23	17 h	0

14	0.25	1:5(Pen17zyme1B):0	20% PEG 8000	15	23	17 h	0

15	0.25	1:5(Pen17zyme1B):0	500 μM spermine	15	23	17 h	0

16	0.25	1:5(Pen17zyme1B):0	N/A	15	23	17 h	50

17	0.25	1:5(Pen17zyme1B):0	2.2 M potassium acetate	15	23	17 h	0

18	0.25	1:5(Pen17zyme1B):0	3 M LiCl	15	23	17 h	0

19	0.80	1:1(Pen17zyme1B):1	500 μM spermine	15	23	17 h	0

20	0.80	1:10(Pen17zyme1B):1	500 μM spermine	15	23	17 h	0

21	0.80	1:20(Pen17zyme1B):2	500 μM spermine	15	23	17 h	0

22	0.80	1:10(Pen17zyme1B):10	500 μM spermine	15	23	17 h	0

23	0.80	1:10(Pen17zyme1B):10	500 μM spermine	15	23	17 h	6

24	0.80	1:10(Pen17zyme1B):10	500 μM spermine	15	23	40 h	0

25	0.80	1:10(Pen17zyme1B):10	500 μM spermine	15	23	72 h	0

26	0.80	1:10(bioPen17zyme1B):10(biopen17zyme2)	500 μM spermine	15	23	72 h	0

* The value corresponds to the final concentration of the indicated component in the reaction mixture.

### Isolation of 5'-biotinylated DNAzymes and RNA Fragments by Affinity Capture

Biotinylated DNAzymes were removed from the mixture of cleavage reaction products using streptavidin agarose beads (Molecular Probes, Eugene, OR) in 3× NTE buffer (0.6 M NaCl, 15 mM Tris-HCl, 7.5 mM EDTA, pH 7.5) [47]. The DNAzymes were recovered from the beads by heating in H_2_O at 65°C. The excised RNA fragments were isolated by affinity capture through hybridization to a complementary oligodeoxyribonucleotide, bioantiPEN (Table 1), immobilized on streptavidin agarose beads via streptavidin-biotin linkage [47].

## Results and Discussion

### Expression of 3×*pen *aRNA in strain JM109 (DE3)

**Figure 2:**
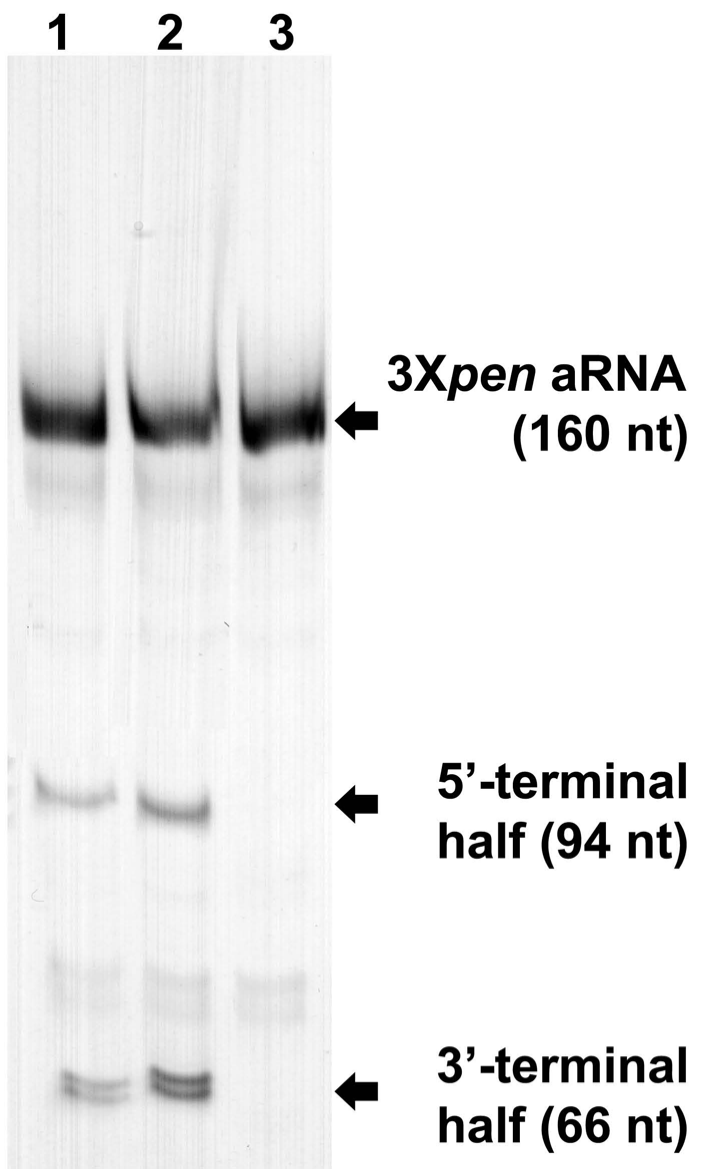
**3×*pen *aRNA cleavage by Pen17zyme1, Pen17zyme1B or Pen17zyme1C**. Cleavage reaction products were analyzed on denaturing 8% PAGE. The expected cleavage products are 94-nt 5' terminal and 66-nt 3' terminal halves of 160-nt 3×*pen *aRNA. Lane 1, 3×*pen *aRNA cleavage with Pen17zyme1; Lane 2, 3×*pen *aRNA cleavage with Pen17zyme1B; Lane 3, 3×*pen *aRNA cleavage with Pen17zyme 1C. Cleavage reactions were set up as follows: RNA substrate and indicated DNAzyme were annealed in 50 mM MOPS-NaOH (pH 7.2), 500 μM spermine by incubating the mixture at 90°C for 2 min followed by cooling to 23°C within 10 min. After annealing, the composition of the mixture was adjusted to 125 mM KCl, 500 mM NaCl, 7.5 mM MgCl_2_, 15 mM MnCl_2_, 150 μM spermine, and 50 mM MOPS-NaOH (pH 7.2), with 3×*pen *aRNA: DNAzyme molar ratio 1: 5. The reaction was performed at 23°C for 16 hours, and stopped by ethanol precipitation of the products.

**Figure 3:**
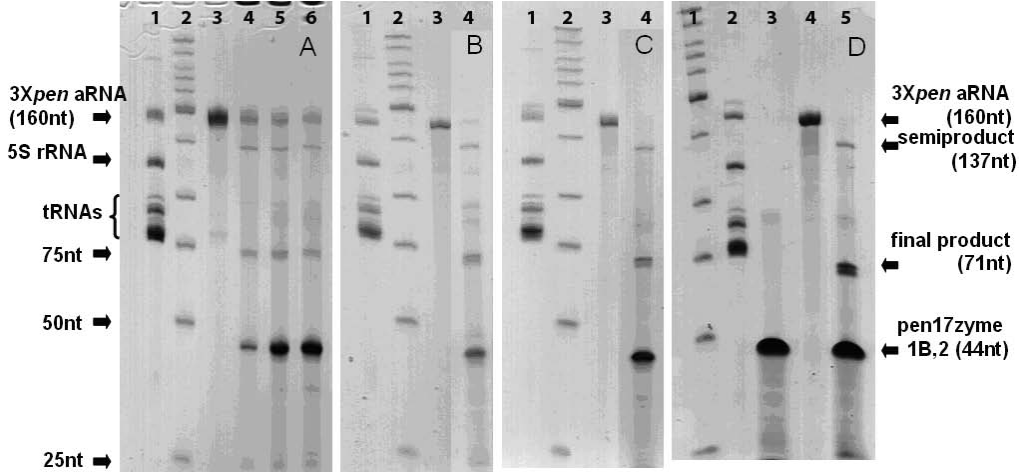
**3×*pen *aRNA cleavage by Pen17zyme1B and Pen17zyme2**. Cleavage reaction products were analyzed on denaturing 8% PAGE. Incubation of 3×*pen *aRNA (160 nt) with the Pen17zyme1B and Pen17zyme2 resulted in accumulation of 71-nt final excision product and 137-nt semi-product. (A) Lane 1, total RNA from *E. coli *JM109(DE3)/pCP3×3 enriched for low molecular weight RNAs (total RNA); Lane 2, low molecular weight DNA ladder (LWM); Lane 3, 3×*pen *aRNA; Lanes 4-6, Cleavage products of 3×*pen *aRNA after 17 hours of incubation with 3×*pen *aRNA: Pen17zyme1B: Pen17zyme2 molar ratio 1:1:1, 1:10:1, and 1:10:2, respectively. (B) Lane 1, total RNA; Lane 2, LWM; Lane 3, 3×*pen *aRNA; Lane 4, Cleavage products of 3×*pen *aRNA after 40 hours of incubation with 3×*pen *aRNA: Pen17zyme1B: Pen17zyme2 molar ratio 1:10:1. (C): Lane 1, total RNA; Lane 2, LWM; Lane 3, 3×*pen *aRNA; Lane 4, Cleavage products of 3×*pen *aRNA after 40 hours of incubation with 3×*pen *aRNA: Pen17zyme1B: Pen17zyme2 molar ratio 1:10:10. (D): Lane 1, LWM; Lane 2, total RNA; Lane 3, equimolar mixture of Pen17zyme1B and Pen17zyme2; Lane 4, 3×*pen *aRNA; Lane 5, Cleavage products of 3×*pen *aRNA after 72 hours of incubation with 3×*pen *aRNA: Pen17zyme1B: Pen17zyme2 molar ratio 1:10:10. Cleavage reactions were performed under conditions described in legend to Figure 2 except for the incubation time and 3×*pen *aRNA: DNazymes molar ratio, which were as indicated above.

The 160-nt 3×*pen *aRNA was strongly expressed in *E. coli *(Figure 2 and Figure 3A, Lane 1). The ratio of 3×*pen *aRNA to 5S rRNA after 8 and 24 h of incubation was 1.07 and 0.46, respectively, as determined by electrophoresis and imaging as described above. Thus, the accumulation of 3×*pen *aRNA after an 8-hour incubation slightly exceeded that of native 5S rRNA but subsequently declined, suggesting selective degradation of the recombinant RNA over time. Isolation of 3×*pen *aRNA from 8-hour bacterial cultures routinely yielded 2.5 - 2.6 mg of the chimeric RNA from 1 g of wet cells.

### Excision of specific small RNA using DNAzymes

**Figure 4:**
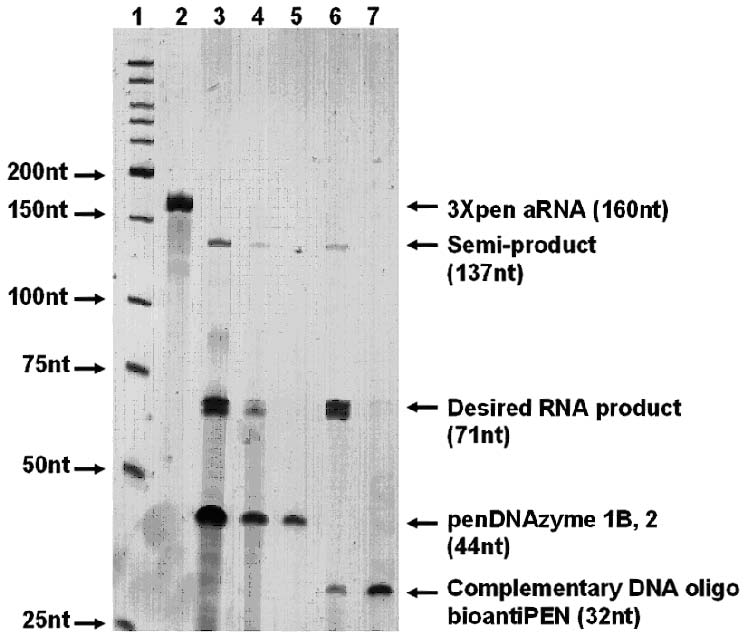
**Separation of the products of 3×*pen *aRNA cleavage using streptavidin agarose beads**. Collected fractions were analyzed on denaturing 8% PAGE. Lane1, LWM; Lane 2, 3×*pen *aRNA; Lane 3, Original cleavage reaction mixture; Lanes 4-5: Isolation of biotinylated DNAzymes, first and second elutions from the beads, respectively; Lane 6: Isolation of 71-nt excision product on the beads saturated with biotinylated complementary deoxyoligonucleotide, bioantiPEN; Lane 7: Recovery of bioantiPEN from the beads. Cleavage reaction was performed for 72 hours under conditions described in legend to Figure 2, with 3×*pen *aRNA: bioPen17zyme1B: bioPen17zyme2 molar ratio 1:10:10.

In order to optimize the cleavage reaction, multiple reaction conditions were examined (Table 2) by varying DNAzymes, RNA substrate to DNAzymes ratio, additives, Mn^2+ ^concentration, reaction temperature, and incubation time. In some cases, alternating denaturation and renaturation cycles were introduced to examine whether this increased the overall yield of the desired RNA products. Equal amounts of 3×*pen *aRNA were used in each reaction condition, and the reaction products were analyzed by denaturing PAGE. Four DNAzymes (Pen17zyme1, -1B, -1C and -2) were investigated for their ability to excise small RNAs from the 5S rRNA expression scaffold. Their predicted excision sites on the 3×*pen *aRNA are shown in Figure 1, and cleavage at these two sites was expected to produce a 71-nt product. As revealed by electrophoresis, the overall yield of the desired RNA products was higher with Pen17zyme1B than with Pen17zyme1, while Pen17zyme1C was found to be inactive (Figure 2). Thus, Pen17zyme1B, which cleaves GA dinucleotides, and Pen17zyme2, which cleaves GG dinucleotides (Figure 1), were used in subsequent cleavage reactions. Additionally, the 5'-biotinylated DNAzymes were found to cleave with equal efficiency as their unmodified forms, and the expected 71-nt product was obtained likewise by cleavage reactions using 5'-biotinylated bioPen17zyme1B and bioPen17zyme2 (Figure 4, Lane 3). Intensity of the 3×*pen *aRNA band on the polyacrylamide gel (Figure 3) decreased when the concentration of Pen17zyme1B and Pen17zyme2 increased in the reaction mixtures, indicating that 3×*pen *aRNA was being efficiently cleaved by these DNAzymes. Complete excision was achieved after 72 h incubation at 23°C and a molar ratio of [3×*pen *aRNA]: [Pen17zyme1B]: [Pen17zyme2] of 1:10:10 (Table 3).

**Table 3:** Quantitative analysis of total cleavage percentage P of 3×pen aRNA by Pen17zyme1B and Pen17zyme2

Molar ratio of 3×*pen *aRNA: Pen17zyme1B: Pen17zyme2	Reaction time (hours)	71-nt final product yield related to the initial 3×*pen *aRNA amount	137-nt semi-product yield related to the initial 3×*pen *aRNA amount	P (Total cleavage percentage)
1:1:1	17	30%	15%	45%

1:10:1	17	39%	16%	55%

1:10:2	17	42%	15%	57%

1:10:1	40	70%	22%	92%

1:10:10	40	77%	23%	100%

1:10:10	72	90%	10%	100%

Cleavage reactions were performed under conditions described in legend to Figure 2 except for the reaction time and 3×*pen *aRNA: DNazymes molar ratio, which were as indicated above.

In most cases, the presence of additional components in the annealing buffer including ethanol, PEG 8000, potassium acetate, LiCl and increased concentrations of KCl or NaCl did not affect the overall yield of the desired RNA product. However, the addition of 500 μM spermine improved the total cutting percentage significantly. It is thought that the spermine may stabilize the DNAzyme/RNA hybrids [48]. Spermine-mediated stabilization of DNA helices [49] and DNA-RNA hybrids [50,51] has been extensively reported in the literature. Previous studies of DNAzymes have shown that substrate cleavage required divalent metal cations such as Mg^2+^, Ca^2+ ^or Mn^2+ ^[52-55]. Here, the effects of four different Mn^2+ ^conditions (0, 7.5, 15 and 45 mM) were tested. We confirmed that in the absence of Mn^2+^, essentially no cleavage was seen and that the highest total cutting percentage was observed at 15 mM Mn^2+^. With respect to the reaction temperature, it was found that 23°C was superior to 40°C in producing efficient RNA processing (data not shown). Introducing denaturation/renaturation cycles into the reaction did not improve the overall yield of the desired RNA products. Perhaps not surprisingly, it was also found that longer incubation time greatly improved the total cutting percentage when the same concentration of DNAzymes was added in the reaction mixture (Table 3).

In summary, the best conditions among those tested were as follows: (*i*) a substrate to enzyme molar ratio ([3×*pen *aRNA]:[Pen17zyme1B]:[Pen17zyme2]) of 1:10:10, (*ii*) denaturation and annealing in 50 mM MOPS-NaOH (pH 7.2), 500 μM spermine at 90°C for 2 min with subsequent cooling to 23°C over a period of 10 min, (*iii*) incubation in 125 mM KCl, 500 mM NaCl, 7.5 mM MgCl_2_, 15 mM MnCl_2, _150 μM spermine, and 50 mM MOPS-NaOH (pH 7.2), at 23°C for 72 h. As suggested by Table 3, longer incubation time is likely to further improve overall yield of the desired RNA fragment.

### RNA and DNAzyme Recovery by Affinity Capture

It is desirable that the isolation of the RNA product and recovery of DNAzymes avoid labor-intensive procedures. Following the cleavage reaction, the 5'-biotinylated Pen17zymes 1B and -2 were successfully removed from the reaction mixture by affinity chromatography, heat eluted (Figure 4, lane 4 & 5) and could presumably be reused in new cleavage reactions. After removal of the DNAzymes, the reaction mixture was purified by hybridization affinity capture on an immobilized oligonucleotide bioantiPEN, which is complementary to the desired RNA product. Figure 4 illustrates that the RNA eluate from the affinity matrix contained the 71-nt excised RNA fragment, indicating the expected digestion of 3×*pen *aRNA. The RNA product was found to be 82% pure as determined by electrophoresis and imaging as described above. The overall yield of the purified RNA fragment was 0.72 mg from 1 g of cells. However, trace amounts of deoxyoligonucleotide bioantiPEN were found in the RNA elution fraction (Figure 4, lane 6). If desired, this contamination could be easily removed by DNase treatment of the excised RNA fraction. Alternatively, this contamination could be prevented by covalent immobilization of the capture oligo on rigid supports like glass or silica.

## Conclusions

The feasibility of an *E. coli *based *in vivo *expression system capable of producing small RNAs using a 5S rRNA scaffold has been demonstrated. The DNA construct is recognized as essentially a normal RNA coding region and the resulting transcript is processed into a single RNA species of defined sequence and length. The strong expression of chimeric 3×*pen *aRNA here and in nearly twenty other 5S rRNA/insert chimeras examined in previous studies [36,56] demonstrates that the 5S rRNA scaffold is robust and can accommodate many RNA inserts of diverse sizes.

Here, we have also described a new method for total RNA isolation. Compared to traditional RNA isolation methods such as Trizol™ [57,58] or hot phenol extractions, the new method does not involve toxic chemicals. Thus, it is safer to the user, more environmentally friendly, and might be particularly advantageous for scale-up. The new method also displays some degree of desirable size selectivity, in that low molecular weight RNAs appeared to have been enriched, while the amount of larger RNAs had been reduced at the end of the RNA isolation procedure.

In addition, we demonstrated the successful excision of a 3×*pen *aRNA from the scaffold using 8-17 DNAzymes Pen17zyme1B and Pen17zyme2. Since DNAzymes can be readily obtained from various commercial sources, and the reaction preferentially occurs at room temperature (23°C), it constitutes an attractive method for the large scale preparation of small RNAs through fermentation of *E. coli*. Under best reaction conditions, nearly all the 3×*pen *aRNA was digested, and 90% of it was further processed to yield the desired 71-nt RNA product after 72 h. Depending on the requirements, the rate of cleavage can be manipulated by adjusting the DNAzyme-to-RNA ratio to either speed up the process or conserve DNAzymes. In an industrial environment, the priority might be given to reducing the costs of reagents *vs*. the reaction rate. Manufacturing processes usually involve continuous operations, and under such circumstances the time for individual reaction cycles may not be as important as the cost for labor, chemicals, and equipment.

In a further step towards improving the economical attractiveness of the method, we have shown recovery of both the RNA product and the DNAzymes through affinity chromatography. Although DNA oligomers can be chemically synthesized, the ability to readily recycle the DNAzymes will substantially reduce the cost of the whole procedure. In the present study, affinity chromatography of streptavidin agarose beads was employed to demonstrate the possibility of recovering DNAzymes. Future studies may include covalent immobilization of DNAzymes on more durable and less expensive supports such as glass or silica, which would be more compatible with industrial applications. In addition, it was shown that a longer incubation time greatly improved the overall yield of desired RNA product with equal concentration of DNAzymes in the reaction mixtures. Therefore, the cost of DNAzymes can be further reduced by trading off speed for cost.

In summary, a fermentation-based approach to large scale RNA production of functional RNAs using the 5S rRNA scaffold strategy is described. The approach combines high levels of *in vivo *expression, convenient purification of chimeric RNAs and cost-efficient excision of insert RNA from the chimera and therefore offers a promising alternative for large scale RNA manufacturing.

## List of abbreviations

RNA: ribonucleic acid; DNA: deoxyribonucleic acid; 5S rRNA: 5S ribosomal RNA; mRNA: messenger RNA; tRNA: transfer RNA; DNAzyme: deoxyribozyme; RNAi: RNA interference; PEG: polyethylene glycol; PAGE: polyacrylamide gel electrophoresis; UV: ultraviolet; TLC: thin-layer chromatography; EDTA: ethylenediaminetetraacetic acid; HEPES: N-2-hydroxyethylpiperazine-N'-2-ethanesulfonic acid; MOPS: 3-(N-morpholino)propanesulfonic acid.

## Competing interests

GEF, VGS, GWJ, and YL have one or more pending patent applications on the described technology.  This intellectual property has been licensed to BioTex, Inc.

## Authors' contributions

GEF, VGS and GWJ conceived the study. YL and VGS designed and performed the experiments. US, RCW, and GWJ participated in data analysis. All authors contributed to drafting the manuscript. All authors read and approved the final manuscript.
